# One for All or All for One: Heterogeneous Expression and Host Cell Lysis Are Key to Gene Transfer Agent Activity in *Rhodobacter capsulatus*

**DOI:** 10.1371/journal.pone.0043772

**Published:** 2012-08-20

**Authors:** Paul C. M. Fogg, Alexander B. Westbye, J. Thomas Beatty

**Affiliations:** Department of Microbiology & Immunology, University of British Columbia, Vancouver, British Columbia, Canada; Queen's University, Canada

## Abstract

The gene transfer agent (RcGTA) of *Rhodobacter capsulatus* is the model for a family of novel bacteriophage-related genetic elements that carry out lateral transfer of essentially random host DNA. Genuine and putative gene transfer agents have been discovered in diverse genera and are becoming recognized as potentially an important source of genetic exchange and microbial evolution in the oceans. Despite being discovered over 30 years ago, little is known about many essential aspects of RcGTA biology. Here, we validate the use of direct fluorescence reporter constructs, which express the red fluorescent protein mCherry in *R. capsulatus*. A construct containing the RcGTA promoter fused to mCherry was used to examine the single-cell expression profiles of wild type and RcGTA overproducer *R. capsulatus* populations, under different growth conditions and growth phases. The majority of RcGTA production clearly arises from a small, distinct sub-set of the population in the wild type strain and a larger sub-set in the overproducer. The most likely RcGTA release mechanism concomitant with this expression pattern is host cell lysis and we present direct evidence for the release of an intracellular enzyme accompanying RcGTA release. RcGTA ORF *s* is annotated as a ‘cell wall peptidase’ but we rule out a role in host lysis and propose an alternative function as a key contributor to RcGTA invasion of a target cell during infection.

## Introduction

*Rhodobacter capsulatus* is a photosynthetic α-proteobacterium [1], [2] and the model organism for the study of an unusual class of mobile genetic elements known as gene transfer agents (GTAs) [3], which have been implicated as important vectors of widespread lateral gene transfer in the oceans [4]. A variety of functional GTAs have been described from diverse prokaryotic species such as *Brachyspira hyodysenteriae* [5], *Desulfovibrio desulfuricans* [6], *Methanococcus voltae* [7], *Reugeria pomeroyi* [8] and *Bartonella grahamii* [9], [10], however, the GTA of *R. capsulatus* (RcGTA) is an archetype of a class of related elements and remnant genes that is exclusive to the α-proteobacteria [11], [12]. The RcGTA genes and homologues exhibit a great degree of synteny, and appear to have descended from a common ancestor by vertical inheritance [11], [13]. RcGTA and related sequences in the α-proteobacteria are thought to have evolved from a defunct ancient prophage that has been hijacked by the bacterial host to perform a new function, and is now so integrated as to be indistinguishable from other native cellular elements [13]. Whilst this theory is by no means incontrovertible, it is the most reasonable explanation based on current data.

Morphologically, RcGTA is a small, tailed phage-like particle that packages and transfers host DNA [14] to closely related cells with no clear bias for any specific genes, including those that encode the GTA. Furthermore, the quantity of DNA packaged by all GTAs that have so far been characterized (4–14 kb) is insufficient to mobilize all of the genes required for GTA production [11], [15]. Therefore, GTAs seem to be a mechanism for non-selective lateral transfer of genes within a population. RcGTA particles begin to accumulate during early stationary growth phase, when grown in complex media, and are released into the supernatant shortly thereafter [16]. However, the method by which the RcGTA particles are released from the cell has yet to be determined, although two alternative methods have been proposed [11], [16]. The first is that there is a continuous shedding of viral particles, perhaps akin to the filamentous phage M13 [17], although this method of release has never been described for a tailed-phage. The alternative proposal is a conventional phage-like lytic burst from a sub-population, and extrapolation of bioassay data suggested that lysis of only 10^5^–10^6^ cells/ml could account for the RcGTA levels observed in the wild type *R. capsulatus* B10 strain [16]. There is a precedent for this as the *B. hyodysenteriae* GTA, VSH-1, encodes a putative holin and an experimentally-confirmed endolysin, similar to that of *Salmonella* phage epsilon15 [18]. However, there are no proteins encoded by the RcGTA cluster that are clear homologues to characterized lytic enzymes.

Random mutation of the *R capsulatus* genome by treatment with nitrosoguanidine has produced an RcGTA overproducer strain, Y262, with RcGTA titres in the region of 1000-fold greater than strain SB1003 [14]. Subsequent work derived additional mutant strains, R121 [19] and DE442, from Y262 that surpassed the levels of RcGTA produced by the parent. Here we examined the RcGTA expression profiles of wild type and overproducer *R. capsulatus* cultures (SB1003 and DE442, respectively) under different growth conditions and on a single cell level. We also investigated whether lysis could be the mechanism of release and whether a gene within the RcGTA cluster could be responsible for release.

## Materials and Methods

### Bacterial Strains and Growth Conditions

All western and fluorescence experiments were carried out on the sequenced *R. capsulatus* strain SB1003 [20] and the overproducer strain DE442, derived from Y262 [14] (Table 1). Bioassays for RcGTA produced by SB1003 and DE442 were carried out using the rifampicin sensitive wild type strain B10 as the recipient of the rifampicin resistance marker [2] (Table 1). *R. capsulatus* strains were cultured in 80% full flasks agitated at 150 rpm to achieve low aeration conditions [21], or in completely filled, sealed vessels for anaerobic (photosynthetic) growth with incandescent lamp illumination of ∼100 µM^.^m^−2.^s^−1^. For all experiments, cultures were grown at 30–33 °C in either RCV minimal medium [22] or YPS complex medium [23], and samples were harvested at logarithmic (16 h) and stationary growth phase (40 h) time points. The flow cytometry and fluorescence microscopy results presented are representative experiments which were repeated on three independent occasions. Malate dehydrogenase and absorption spectra were obtained from a total of 4 samples on two independent occasions.

**Table 1:** Bacterial strains and plasmid constructs used in this study.

Strain or Plasmid	Reference or Source	Description
SB1003	[57]	*R. capsulatus* wild type, Rif^R^
B10	[2]	*R. capsulatus* wild type, Rif^S^
DE442	?^a^	*R. capsulatus* RcGTA overproducer, Rif^R^
Y262	[14]	*R. capsulatus* RcGTA overproducer, Rif^R^
BL21 Star	Novagen (Merck KGaA, Germany)	*E. coli*, Protein over-expression strain
pUCpG	This work	pUC18, GTA promoter, Amp^R^
pUCpP	This work	pUC18, *puf* promoter, Amp^R^
pmCherry	Clonetech (http://www.clontech.com/)	pUC, mCherry red fluorescence protein, Amp^R^
pUCpGmC	This work	pUC18, GTA promoter, *mCherry*, Amp^R^
pUCpPmC	This work	pUC18, *puf* promoter, *mCherry*, Amp^R^
pRK415	[29]	Broad host range vector, Tet^R^
pGmC	This work	pRK415, GTA promoter, *mCherry*, Tet^R^
pPmC	This work	pRK415, *puf* promoter, *mCherry*, Tet^R^
pZJD29a	J. Jiang and C.E. Bauer, unpublished	Suicide plasmid, *sacB*, Gent^R^
pUCΔg14	This work	pUC18, ΔRcGTAg14, Amp^R^
pET28a	Novagen (Merck KGaA, Germany)	Expression vector, Kan^R^
pETg14.1	This work	pET28a, RcGTAg14, *lacI*, T7 promoter, Kan^R^, Clone 1
pETg14.2	This work	pET28a, RcGTAg14, *lacI*, T7 promoter, Kan^R^, Clone 2

a Of uncertain provenance, a *crtD* mutant probably derived from Y262 (B. Marrs, personal communication).

**Figure 1:**
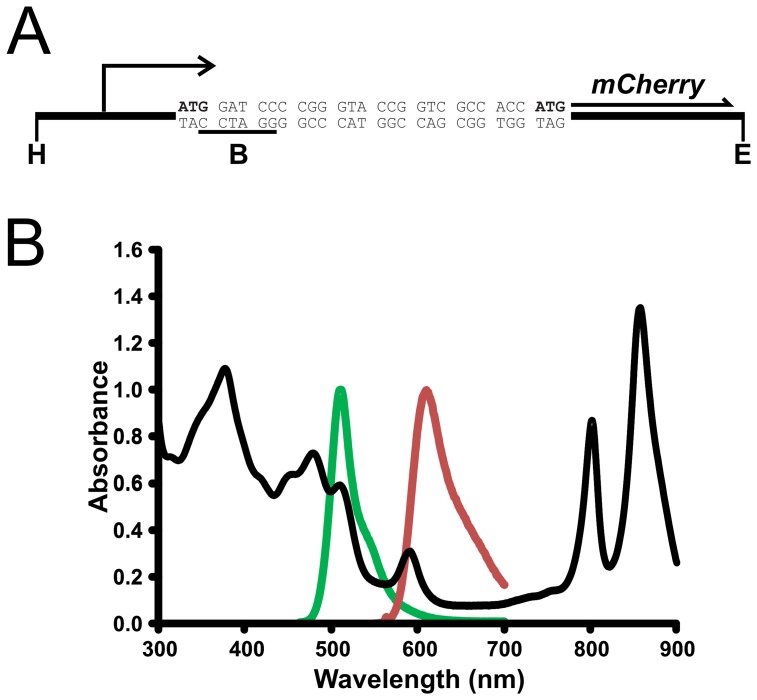
Reporter plasmid promoter fusions to the *mCherry* gene, and comparison of emission from of fluorescent proteins to *R. capsulatus* absorption wavelengths. A. Representation of the *puf* operon and RcGTA promoter regions fused to the *mCherry* gene, showing the fluorescent protein modified N-terminal coding sequence translated from the *pufB* or RcGTA *g1* start codon. The bent arrow indicates either the *puf* operon or RcGTA promoter. The start codons of the *pufB*, RcGTA *g1*, and *mCherry* genes are shown in bold, separated by eight codons from the plasmid pmCherry multiple cloning site. Positions of restriction sites introduced are indicated as Hind III (**H**), BamH I (**B, and sequence underlined**) and EcoR I (**E**). **B.** A broad wavelength absorbance scan of *R. capsulatus* SB1003 is shown as a black trace. The emission spectra of eGFP and mCherry are represented as green and red lines, respectively.

**Figure 2:**
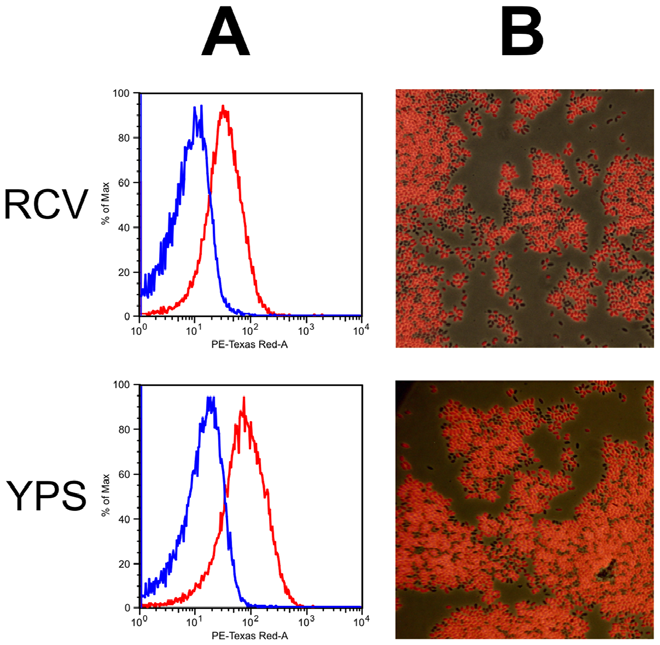
Fluorescence emitted from *puf* promoter reporter gene fusion cultures. **A.** Flow cytometry plots of emission at 610 nm from SB1003 cultures on the X-axis against cell counts as percentage of the maximum on the Y-axis. Cultures were grown to stationary phase in either YPS complex medium (upper panel) or RCV minimal medium (lower panel). The negative control data (cells lacking plasmid pPmC); blue) were overlaid with experimental data (cells containing plasmid pPmC; red). Graphs shown are single representative experiments. **B.** Fluorescence microscopy of the same cultures. Photographs of cells illuminated with white light were overlaid with fluorescence images of the same microscope field.

**Figure 3:**
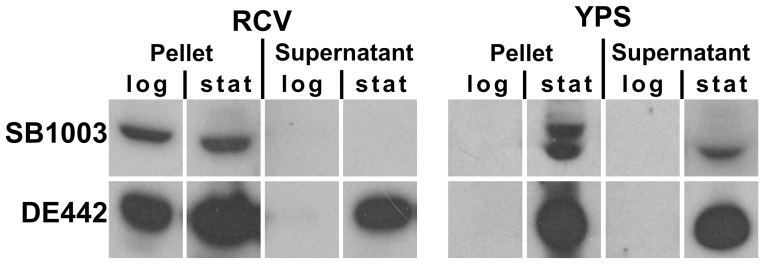
Western blots of the RcGTA capsid protein. Pellets and supernatants from WT SB1003 and DE442 overproducer cultures grown in complex (**YPS**) or minimal (**RCV**) media, harvested during logarithmic (**log**) and stationary (**stat**) growth phases and probed with an RcGTA capsid antiserum.

### Western Blots

Cultures were grown as described above, cells were harvested by centrifugation and supernatants were filtered through a 0.2 μm pore-size filter. Samples were normalized to the equivalent of a 1 ml culture with an OD_660_ reading of 1. Samples from the wild type strain were concentrated 10-fold and heated to 95°C for 10 min in 1 × Laemmli buffer [24], whereas those from the overproducer strain were unconcentrated but otherwise treated identically to the wild type. Samples were run on 12% polyacrylamide gels [24] in a Mini-PROTEAN II system (Bio-Rad, CA, USA), according to the manufacturer's guidelines. Proteins were transferred onto a BioTrace NT nitrocellulose membrane (Pall Life Sciences, NY, USA), using a Mini Trans-Blot apparatus (Bio-Rad) in electroblot buffer (27.5 mM Tris-base, 192 mM glycine and 20% methanol) at 30 V for 16 h. The membranes were first probed with a rabbit anti-capsid antiserum raised against the RcGTA capsid protein, followed by an HRP-conjugated donkey anti-rabbit secondary antibody. Detection was carried out using the Immobilon western chemiluminescent HRP substrate, according to the manufacturer's instructions (EMD Millipore, MA, USA). Samples for size exclusion experiments were passed through an Amicon centrifugal filter unit with a 100 kDa cut-off (EMD Millipore). Both the flow-through and the rententate (diluted to the original volume) were then analyzed by western blotting as described above.

**Figure 4:**
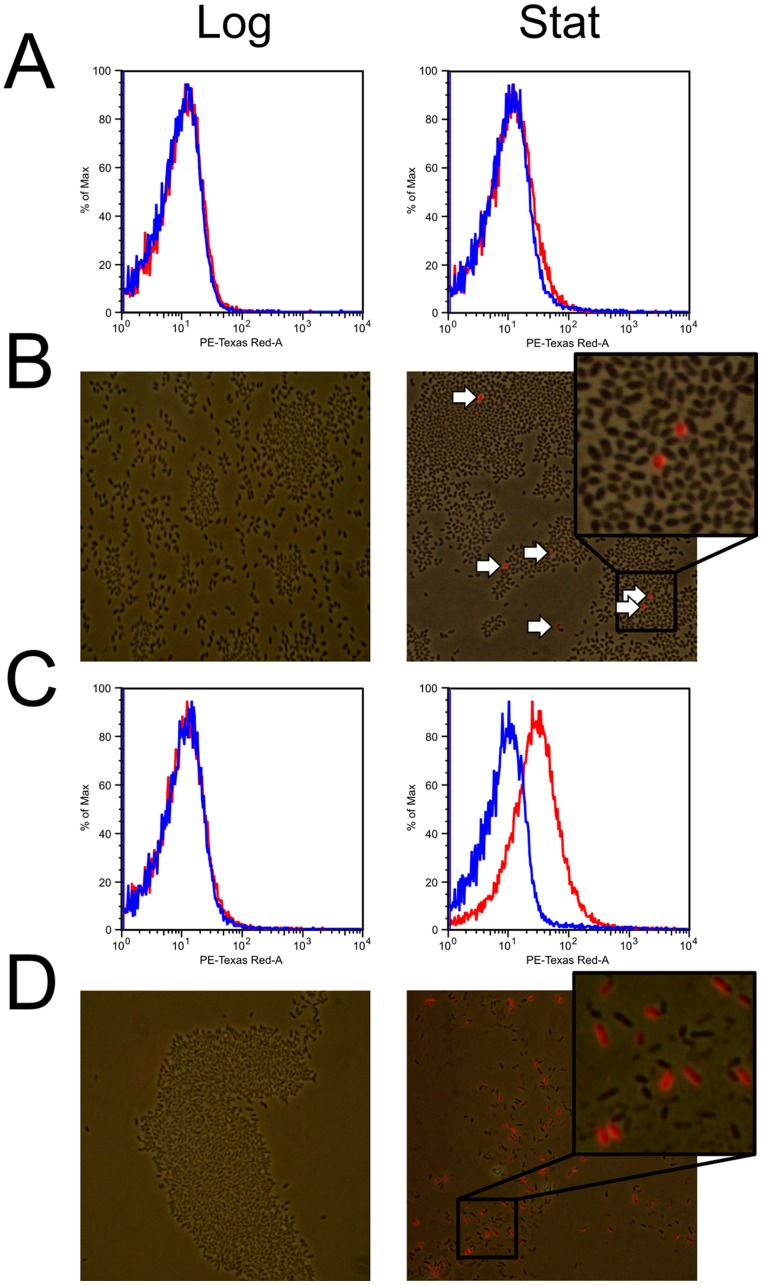
Fluorescence emitted from GTA promoter reporter gene fusion cultures. Flow cytometry plots of emission at 610 nm on the X-axis against cell counts as percentage of the maximum on the Y-axis. All Cultures were grown to either logarithmic (**Log**, 16 h) or stationary phase (**Stat**, 40 h) in complex media. The negative control data (blue) were overlaid with experimental data (red). Graphs shown are single representative experiments. **A.** SB1003 (pGmC) **C.** DE442 (pGmC). **Fluorescence Microscopy.** Photographs of cells illuminated with white light were overlaid with isogenic fluorescence images. **B.** SB1003 (pGmC). **D.** DE442 (pGmC).

**Table 2:** Quantitative data of single cell fluorescence measurements.

	Flow Cytometry				
	Mean	FoP (%)	SEM (%)	n	Σ cells
SB1003	11.00	0.81	0.13	3	90,000
SB1003 (pGmC)	12.00	0.95	0.15	3	90,000
DE442	9.90	1.36	0.13	3	90,000
DE442 (pGmC)	39.50	37.45	0.32	3	90,000

	Fluorescence Microscopy				
	Positive Cells	Proportion (%)	SEM (%)	n	Σ cells
SB1003	0	0	0	3	N/A*
SB1003 (pGmC)	54	0.15	0.03	3	35,911
DE442	0	0	0	3	N/A*
DE442 (pGmC)	1,330	26.39	2.60	3	5,040

Mean: Fluorescence in arbitrary units; FoP: Frequency of Parent; SEM: Standard Error of the Mean; n: number of independent experiments; Σ cells: the total number of cells within those experiments.

N/A*: No total cell counts were recorded for negative controls as no cells were fluorescent.

### Fluorescence construct

The RcGTA promoter was amplified using primers with incorporated Pst I and BamH I restriction sites, pGTA5 and pGTA2.6 [25]. The *puf* (photosynthesis gene) operon promoter [26] was amplified using primers with incorporated Hind III and BamH I restriction sites (puf F: 5′-GACGGCAAGCTTCGGAATCTGCG-3′; puf R: 5′-CTTAGGATCCATAACAACCTCCGGATTG-3′). Both promoters were cloned into pUC18 to form pUCpG and pUCpP, respectively. The mCherry gene [27], [28] was excised from pmCherry (Table 1) using BamH I and EcoR I restriction endonucleases and cloned into pUCpG and pUCpP (Table 1). In the resultant constructs, the mCherry start codon is directly preceded by eight codons from the pmCherry plasmid and then the start codon and Shine-Dalgarno sequence of the RcGTA *orf1* and *pufB* genes, respectively (Table 1). The two resulting promoter-mCherry fusions were excised with Hind III and EcoR I and transferred to the broad host range plasmid pRK415 [29] to produce the pGmC (RcGTA promoter driving transcription of the mCherry coding region) and pPmC (the *puf* operon promoter driving transcription of the mCherry coding region) constructs (Table 1).

**Figure 5:**
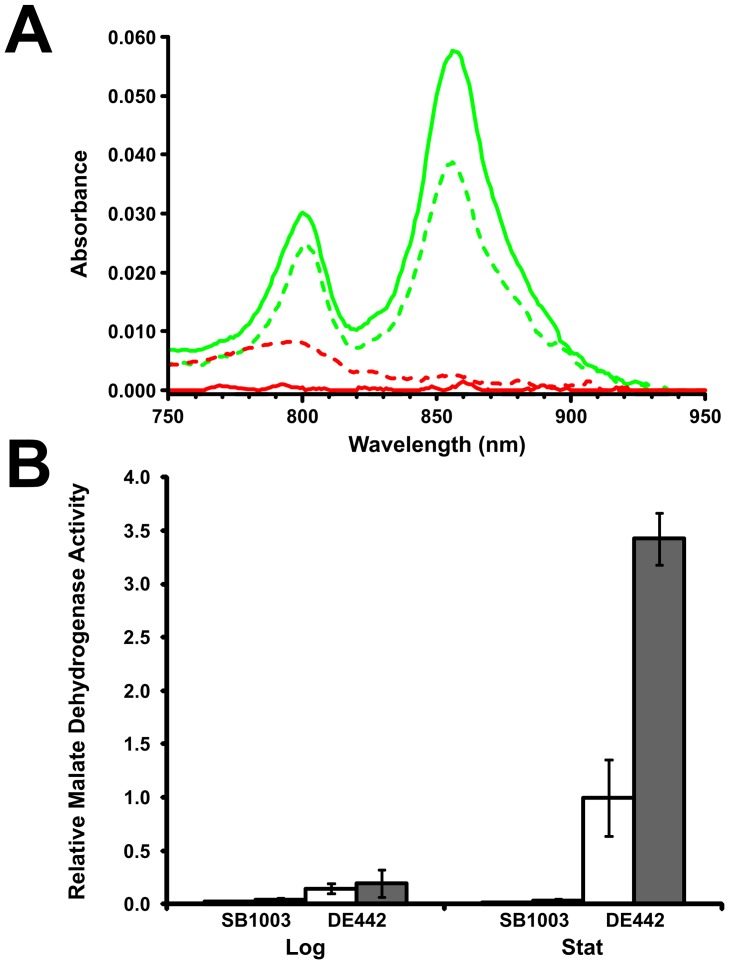
Detection of lysis in *R. capsulatus* wild type and GTA overproducer strains. A. The absorption spectra of cell-free supernatants from stationary cultures were recorded from 1000 nm to 250 nm to determine the level of LH2 pigment release to supernatant. The region from 750 nm to 950 nm shows characteristic LH2 bacteriochlorophyll peaks at 802 nm and 855 nm for the overproducer strain DE442 (green) and the wild type strain SB1003 (red). Cultures were grown in complex (solid lines) or minimal medium (dashed lines). **B.** Activity of the citric acid cycle enzyme malate dehydrogenase was determined in cell-free supernatant and cell-free French pressed whole culture by measuring the decrease in OD at 340 nm. Samples were harvested from wild type (**SB1003**) and GTA overproducer (**DE442**) strains at logarithmic (16 h, **Log**) and stationary (40 h, **Stat**) growth phases. Cultures grown in RCV minimal medium are depicted as open bars and those grown in YPS complex medium by closed bars. Mean ratios of supernatant to whole culture were calculated for a total of 4 samples from 2 independent experiments. Error bars represent standard deviation.

### Flow cytometry

For each sample, 1 ml of cells was harvested by centrifugation and resuspended in 3 ml PBS buffer (137 mM NaCl, 2.7 mM KCl, 10 mM Na_2_HPO_4_, 2 mM KH_2_PO_4_, pH 7.4). Fluorescence emission from these live, unfixed samples was analysed on an LSRII analyser equipped with a 561 nm laser line (BD Biosciences, ON, Canada). Sub-optimal 561 nm excitation should achieve ∼60% of maximum efficiency. Emission spectra were passed through a 610 nm (+/−10 nm) filter. 30,000 fluorescence events were recorded per sample and data were graphically represented using FlowJo (www.flowjo.com). Pacific Blue (Ex. Max: 401 nm/Em. Max: 452 nm) and PerCP (Ex. Max: 482 nm/Em. Max: 678 nm) were used as a control readings for non-specific auto-fluorescence, as the excitation and emission spectra do not overlap with the mCherry excitation maximum of 587 nm and emission maximum of 610 nm. Laser intensity was empirically defined for each experiment such that the negative control trace peaked at a fluorescence intensity of ∼10^1^ and abolished at ∼10^2^. All samples were gated for forward and side scatter, to ensure measurements were predominantly of cells, and additionally for fluorescence in excess of basal levels. The basal threshold was set to exclude 99% of negative control cells, above this threshold was designated frequency of parent (FoP). The negative control cultures for fluorescence experiments were the relevant host strains in the absence of the reporter plasmid.

**Figure 6:**
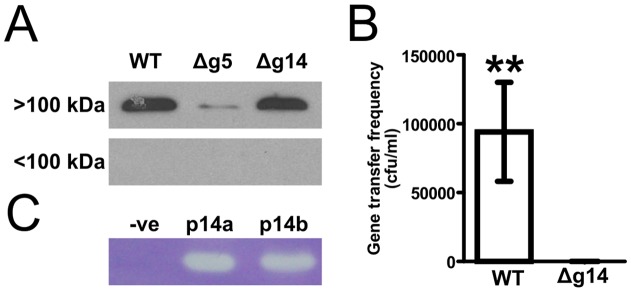
Role of p14 in RcGTA release. **A.** Wild type *R. capsulatus* SB1003 culture supernatant fractions were prepared by size exclusion filtration, and used in western blots probed with RcGTA capsid antiserum. Blots are shown of the fraction containing proteins in excess of 100 kDa (**Upper Panel**) and that containing proteins less than 100 kDa (**Lower Panel**). The designation Δg5 indicates the supernatant from a transposon knock-out of RcGTA *g5*, used as a negative control strain that lacks the capsid protein. **B.** Gene transfer frequency for SB1003 and SB1003Δg14. Strains were grown anaerobically with illumination to stationary growth phase in YPS. Frequency of gene transfer is the number rifampicin resistant colonies per ml, resulting from infection of a rifampicin-sensitive strain (B10) with RcGTA particles from the rifampicin resistant strains SB1003 or SB1003Δg14. Means were calculated from 3 biological replicates. Error bars show standard deviation. Double asterisks denote a p-value ≤0.01 derived from a one tailed, two sample t-test. **C.** Peptidoglycan zymogram of two *E. coli* pET28a-*g14* overexpression cultures (**p14a/b**) and a negative control (**−ve**) of *E. coli* lacking the *g14* gene. Gels were stained with Coomassie brilliant blue and zones of clearing indicate localized degradation of peptidoglycan.

### Fluorescence microscopy

Images were taken using a Sony DSC-S75 digital camera mounted on a Zeiss Axioskop fluorescent microscope at 100× magnification. Excitation was achieved using a mercury lamp (HBO 50W) and Zeiss filter set 15 (excitation: 546 nm/12 nm; emission: 590 nm). Total cell counts and the fraction of fluorescent cells were calculated using the National Institute for Health image processing software, ImageJ [30].

### Malate dehydrogenase assay

Cell-free culture supernatants were obtained by centrifugation at 16,000×g followed by filtration of the supernatant through a 0.2 µm pore filter. Total lysate samples were obtained by passing whole cultures through a French pressure mini cell (Aminco) followed by centrifugation at 13,500× g to remove cellular debris. Samples of 100 µl were mixed with 0.2 mM NADH and 100 mM KPO_4_ (pH 7.4) in a total volume of 980 µl. The assay was initiated by addition of 20 µl of 10 mM oxaloacetic acid, and the decrease in absorbance at OD_340_ over time was measured in a U-3010 Spectrophotometer (Hitachi). The Δ_A340_/min was calculated from the initial linear portion of the curve using the Time Scan function of UV Solutions 2.1 (Hitachi) software. Activity was calculated in units/ml using the equation: 
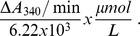


### Absorption spectroscopy

A wavelength scan from 1000 nm to 250 nm was performed on cell-free culture supernatants and French press-lysed cultures using a U-3010 Spectrophotometer (Hitachi) and UV Solutions 2.1 (Hitachi). The curves were smoothed using the 7-points mean function in UV Solutions.

### Knock-out construction

RcGTA g14 flanking regions of ∼500 bp were amplified using two sets of primer pairs (g14 LF: 5′-GCACCAAGCTTTCGGTCGACAATACCG-3′/LR: 5′-CGACCTGCAGCATCGAAACAGGCTCC-3′; g14 RF: 5′-ATGTCTGCAGTGAATGGCGACGATACTG-3′/RR: 5′-CTGCGGATCCTCCAGCACCACATAGG-3′) and cloned sequentially into pUC18 using the underlined restriction sites (Hind III, Pst I, BamH I) (Table 1). The two concatenated fragments were then excised (BamH I and Hind III) and sub-cloned into the suicide plasmid pZJD29a) (Table 1). Acquisition of gentamycin (3 µg/ml) resistance indicated a single crossover event and resistant colonies were cultured aerobically for 3 days in the absence of antibiotic selection. Loss of the suicide plasmid was detected by counter-selection facilitated by the *sacB* conditionally lethal gene after growth on YPS agar +10 % sucrose. Gene deletion was confirmed by PCR using the g14 LF/RR primers (see above).

### Gene transfer assay

GTA activity was measured essentially as previously described [16]. In brief, rifampicin sensitive recipient strains and rifampicin resistant donor strains (Table 1) were grown in RCV and YPS broth, respectively, to stationary growth phase. Recipient cells were harvested by centrifugation and resuspended in 0.4 vol. G-buffer (10 mM Tris pH 8.0, 1 mM MgCl_2_, 1 mM CaCl_2_, 1 mM NaCl, 500 μg/ml BSA Fraction V). Donor cells were removed by centrifugation and discarded, the supernatant was filtered through a 0.2 µm pore-size filter and diluted in G-buffer when required. 100 µl donor filtrate, 100 µl recipient cells and 400 µl G-buffer were combined in 5 ml polystyrene tubes and incubated at 30°C with shaking (200 rpm). After 90 min, 900 µl of RCV medium was added with further incubation for 4 h. Cells were harvested, resuspended in 100 µl RCV and plated onto RCV agar containing 80 µg/µl rifampicin. Gene transfer frequency was normalised to culture density by measuring turbidity at 660 nm.

### Zymogram

Cells from a 1 litre stationary phase, wild type strain SB1003 photosynthetic culture were harvested by centrifugation and resuspended in 100 ml of 4% SDS. The cells were then boiled vigorously for 15 min and the insoluble material was recovered by centrifugation at 10,000×g for 10 min, and the pellet was washed 5 times with 10 ml of dH_2_O [31]. SDS-PAGE gels were prepared, consisting of a 4% (w/v) stacking layer and a 12% (w/v) resolving layer with 5% (w/v) crude peptidoglycan incorporated.

The RcGTA g14 gene was amplified using a primer pair (g14 F: 5′-TCAATCATATGAGCGCGGTCGGGCTGC-3′ and R: 5′-TCACTGAATTCGAAAGGCCCAGAACC-3′) with restriction sites incorporated (Nde I and EcoR I). The amplicon was subcloned into pET28a (Table 1). Overexpression was carried out in BL21 Star cells (Table 1) by addition of 1 mM IPTG to mid-logarithmic growth phase cultures (A_600_: 0.5). The negative control was BL21 Star(pET28a) cells treated identically. 1 ml samples were harvested after 3 h, concentrated 10-fold and heated to 95°C for 10 min in 1 × Laemmli buffer [24]. Samples were run at 70 V for ∼2 h. To renature the proteins, the gels were rinsed with dH_2_O and then washed twice in dH_2_O +5% Triton (v/v) for 20 min. Enzyme incubation were carried out overnight in 100 mM phosphate buffer (pH 7.5) at 37°C with gentle agitation. The gels were stained with Coomassie blue (0.25% w/v) and then destained in a 10% (v/v) acetic acid, 40% (v/v) ethanol solution. Digestion of peptidoglycan produced a zone of clearing against a blue background [32].

## Results and Discussion

### Validation of fluorescence reporter construct

Wild type *R. capsulatus* SB1003 cultures produce RcGTA protein from early stationary phase onwards, and infective particles are subsequently released from the cells under certain conditions [16], [33]. There are no obvious signs of host lysis during this process, such as growth rate impairment or a sudden drop in culture density [16], and there is currently no definitive model for the dynamics of RcGTA expression or mechanism of release [16]. A recent study using a fluorescent lacZ reporter assay reported that the vast majority of RcGTA expression (∼95%) may originate from less than 3% of the population [15]. It is not possible to image live cells using this type of assay as the cells must be permeablized prior to incubation with the substrate, a process which can affect observed fluorescence levels, e.g. because of non-uniform substrate permeation. Here, we describe the production and validation of a direct fluorescence reporter construct, and test whether the differential RcGTA yields observed in SB1003 and the overproducer strain DE442 are indeed a result of a highly active sub-population. To this end, a reporter plasmid (pGmC) was constructed containing the RcGTA promoter fused to the gene for a red fluorescent protein, mCherry (Fig. 1A) [27], [28]. Evidence to date suggests that the RcGTA gene cluster is expressed as a single primary transcript; although microarray data revealed variation in the abundance of individual gene transcript sequences [34], this may be due to segmental differences in mRNA stability. The promoter region chosen here extends from ∼500 bp 5′ of the RcGTA gene cluster to the start codon of the first ORF g1 and, therefore, reflects the activity of the RcGTA promoter mapped in unpublished work [33], [35].

**Figure 7:**
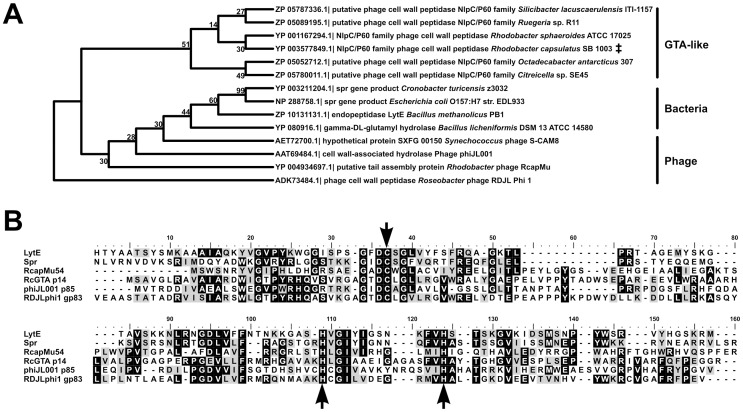
Bioinformatic analysis of RcGTA g14. **A.** Maximum parsimony phylogenetic analysis of the five closest Blastp hits to a p14 query, the two top Spr protein hits, the two top LytE hits, and the four highest scoring bacteriophage hits. Database accession numbers are given on the left, and annotations are on the right. The RcGTA p14 protein is indicated (**‡**). Bootstrap values result from 1,000 iterations. **B.** MUSCLE alignment [56] of RcGTA p14 protein with Spr (*E. coli* O157:H7 EDL933), LytE (*Bacillus methanolicus* PB1), ФJL001 p85, RDJLФ1 gp83 and RcapMu54. Conserved catalytic residues are indicated by arrows. The threshold for shading is 50%; black represents identity and grey represents similarity according to the BLOSUM matrix. Alignment tails with no sequence identity were clipped.

Codon usage for mCherry is optimized for expression in human cells [36] and, although it has been successfully employed in various other species including *Escherichia coli* [37], it has never been tested in *R. capsulatus* (67% GC content) or any member of the α-proteobacteria. Furthermore, in pigmented organisms there is a general issue with signal quenching when using fluorescent proteins that emit light at a wavelength absorbed the pigments, as shown by a superimposition of the eGFP emission spectrum on an *R. capsulatus* absorption spectrum (Fig. 1B). Overlay of the mCherry emission spectrum on an *R. capsulatus* absorption spectrum determined that mCherry is compatible with the endogenous pigmentation because the mCherry peak of fluorescence does not overlap with a major peak of pigment absorption (Fig. 1B).

To empirically test the efficacy of mCherry fluorescence in *R. capsulatus*, the *puf* photosynthesis gene promoter was fused to the mCherry gene to create the plasmid pPmC for use as a positive control (Fig. 1A). RcGTA production and release in *R. capsulatus* has been considered to be optimum under anoxic, photosynthetic growth conditions [16]. However, preliminary experiments using this control mCherry construct yielded no detectable signal under fully anaerobic conditions in *R. capsulatus* SB1003 (data not shown), which is likely to be due to insufficient O_2_ available for maturation of the chromophore [38]. To remedy this problem we used low aeration, chemotrophic growth conditions, under which the *puf* genes are expressed at a high level [21], [26]. Flow cytometry analysis revealed a considerable shift in the population fluorescence of cells harbouring pPmC, compared to the negative control lacking this plasmid. Although there were some minor variations between the different conditions tested, all produced the same general trend (Fig. 2A). These data were supported by fluorescence microscopy, which showed that although there is some variation in the strength of expression from cell to cell, the majority of individual cells produce fluorescence (Fig. 2B). The results were essentially the same regardless of whether cells were cultured in YPS complex or RCV minimal medium. The absence of background fluorescence is indicated by the relatively few dark cells (perhaps due to cells that have lost the plasmid), and more apparent in the experiments using the RcGTA promoter (see below).

To ensure that these low aeration conditions are also appropriate for evaluation of RcGTA production, western immunoblots probed with an RcGTA capsid antiserum were carried out. Considerable capsid accumulation was detected both intracellularly and in the cell-free supernatant, dependent upon the growth conditions, growth phase and strain (Fig. 3). As predicted, in the YPS complex medium there was no appreciable build-up of capsid within the cells during the logarithmic growth phase, and production was up-regulated in the stationary phase along with associated release into the supernatant. The extra band seen in the stationary phase pellet is attributed to a non-capsid cross-reactive protein, as a band at this position was seen using cells of a capsid gene knockout mutant [33]. Unexpectedly, in the RCV minimal medium substantial quantities of intracellular capsid were detected in both logarithmic and stationary growth phases; however, release was not observed for SB1003. The overproducer strain DE442 produced a signal orders of magnitude greater than the wild type strain SB1003, and this massive accumulation was accompanied by the detection of RcGTA, both in the intracellular fraction and supernatant of RCV minimal medium cultures (Fig. 3). These results indicate that RcGTA gene expression is induced under the low aeration, chemotrophic growth conditions employed, although the pattern of RcGTA release differs.

### Single cell RcGTA expression analysis

RcGTA expression profiles were studied by analysis of fluorescence on a single cell level in *R. capsulatus* populations, where cells contained the plasmid pGmC, in which the RcGTA promoter drives transcription of the mCherry coding region (Fig. 1A). No fluorescence was detected in cultures grown in the YPS complex medium during the logarithmic growth phase, when RcGTA production would not be expected (Fig. 4). However, once these cultures moved into the stationary phase of growth, a clear increase in fluorescence was present in all samples (Fig. 4). Flow cytometry of wild type SB1003 stationary phase cells containing plasmid pGmC indicated a slight increase in fluorescence compared to cells lacking the plasmid (Fig. 4A, and Table 2). Subsequent fluorescence microscopy carried out on equivalent samples confirmed that RcGTA is expressed in a very small sub-population of cells (0.15%, Fig. 4B, and Table 2). Flow cytometry of overproducer strain DE442 stationary phase samples revealed that the proportion of plasmid pGmC-containing cells with fluorescence in excess of a gated threshold (Frequency of Parent, FoP values) were significantly greater (27.5-fold, p =  <0.00001) than both DE442 cells lacking the plasmid and the SB1003 wild type strain that contains the plasmid (39.4-fold, p =  <0.00001) (Fig. 4C, Table 2). Fluorescence microscopy broadly corroborated these data, because visible fluorescence was evident in over a quarter of the total population of DE442(pGmC) cells, which corresponds to a 176-fold (p =  <0.00001) increase compared to the wild type strain (Fig. 4D, Table 2). The data yielded by both techniques are consistent with the idea that differential levels of GTA production occur as a result of a sub-population effect, rather than universal modulation of expression across the entire population, as independently concluded by Hynes et al (2012).

### Evidence for lytic release

During the production of cell-free supernatant samples for western blotting, it was apparent that supernatants from the DE442 RcGTA overproducer strain were visibly pigmented under certain conditions. In contrast, comparable supernatants from the SB1003 strain remained relatively clear. To investigate whether RcGTA production correlated with the presence of normally intracellular pigments in the cell-free supernatant, the absorbance of supernatant samples were recorded in a broad spectrum wavelength scan. The light-harvesting 2 (LH2) bacteriochlorophylls of *R. capsulatus* absorb at 802 nm and 855 nm [39], and the absorbance of the larger peak (855 nm) was used for comparison of samples. Supernatant from either wild type SB1003 or overproducer DE442 logarithmic growth phase cultures yielded no detectable peaks corresponding to LH2 (data not presented). However, samples of DE442 stationary phase cultures produced distinct peaks, whereas no peaks were observed for SB1003 (Fig. 5A). The fraction of absorbance at 855 nm in cell-free supernatant to cell-free French-pressed stationary phase cultures of DE442 was calculated to be 5% ± SD 2% (n = 4; grown in YPS complex medium) and 6% ± SD 2% (n = 4; grown in RCV minimal medium).

To obtain an alternative quantitative measurement for the presence of intracellular components in the cell-free supernatant of cultures, we used an enzymatic assay of the citric acid cycle enzyme malate dehydrogenase, and calculated the ratio of activity in cell-free supernatants to cell-free French-pressed whole cultures (Fig. 5B). The wild type strain SB1003 released very low levels of malate dehydrogenase under all of the conditions investigated. The RcGTA overproducer strain DE442 supernatant released a low level of the total malate dehydrogenase activity when harvested after 16 h of growth in either YPS (3% ± SD 2%, n = 4) or RCV (2% ± SD 1%, n = 4) medium. However, the malate dehydrogenase activities present in the supernatants of DE442 cultures harvested after 40 h of growth amounted to a substantial fraction of the total activity in the culture, 45% ± SD 3% (n = 4) in the YPS complex medium, and 11% ± SD 5% (n = 4) in the RCV minimal medium.

Hynes *et* *al.* (2012) reported the presence of a putative lysozyme (rcc00555) and holin (rcc00556) proteins in the *R. capsulatus* SB1003 genome. Disruption of rcc00555 led to a marked decrease in gene transfer activity and intracellular accumulation of intact RcGTA particles, consistent with the proposed function. However, various growth conditions, such as phosphate abundance or carbon limitation [35], [40], and gene disruptions, e.g., *ΔcckA* (unpublished data), have a similar effect on RcGTA release, and no direct evidence for a lytic activity of a protein encoded by either rcc00555 or rcc00556 was presented by Hynes et al (2012). In contrast, our data showing release of normally intracellular pigments and enzyme activity are direct evidence that RcGTA particles escape from a host cell during a lytic event.

### Characterization of a putative cell wall peptidase

In queries of the protein products of RcGTA genes against the NCBI conserved domain database [41], a match of RcGTA orf14 (g14) to the NlpC/P60 family of sugar-binding domains often associated with peptidoglycan hydrolyzing enzymes [42] was revealed. We hypothesised two alternative roles for such a protein in RcGTA biology. Firstly, it is conceivable that degradation of the host cell wall by a member of this family is responsible for lytic release of RcGTA particles [43], [44]. Secondly, such a protein could be required for either recognition of a target receptor or penetration of the cell wall during the process of infection [45].

To test the hypothesis that g14 is required for RcGTA release, a translationally in-frame (silent) deletion of the g14 coding region was created in *R. capsulatus* SB1003. If g14 was essential for RcGTA release, no capsid protein would be detectable in culture supernatant obtained from mutant cultures. However, western blot hybridization revealed that comparable levels of capsid protein accumulated in both wild type and g14 knock-out strains (Fig. 6). Furthermore, centrifugation through size exclusion filters determined that these 32 kDa capsid proteins were accumulated in particles >100 kDa (Fig. 6A), and purification of DNA from cell-free supernatants revealed the presence of characteristic ∼4 kb DNA fragments of DNA present in mature RcGTA particles (data not shown). Although these data do not prove that the intracellular capsid protein was assembled into mature particles, the results indicate that g14 is not involved in RcGTA release.

The majority of RcGTA g14 homologues in genome sequences are annotated as hypothetical proteins or cell wall peptidases, and are found within RcGTA-like gene clusters (Fig. 7A). Beyond these, the p14 protein is distantly related to Spr, an *E. coli* lipoprotein, and LytE, which plays a role in cell wall modification in *Bacillus* sp. [46], [47]. Despite their disparate lineages and <30% amino acid similarity, each protein contains a conserved cysteine peptidase catalytic triad (Cys-His-His), and thus RcGTA p14 is likely to act as a γ-D-glutamyl-L-diamino acid endopeptidase (Fig. 7B) [48]. In addition, NlpC/P60 family proteins are often associated with prophage or prophage-like regions of bacterial genomes [44], [49], [50]. Anantharaman *et* *al.* (2003) proposed that NlpC-like proteins of the Pal lineage are examples of host enzymes that have been subverted by bacteriophages to breach the cell wall during infection. This theory was subsequently applied to the RcGTA g14 homologues found in the Roseophages ΦJL001 and RDJLΦ1 [51], and may also be applicable to a ‘putative tail assembly component’ (rcc00965) encoded by the transposing phage RcapMu, which shares the same *R. capsulatus* SB1003 host as RcGTA (Fig. 7) [52].

To test whether g14 is involved in invasion, RcGTA bioassays were carried out for transfer of rifampicin resistance from the *g14* knockout, compared to the parental strain, to a sensitive host. Despite the fact that RcGTA particles produced by the *Δg14* strain were apparently intact, they were unable to transfer antibiotic resistance to the recipient (Fig. 6B).

To investigate whether the g14 protein is capable of degrading peptidoglycan, lysates of two *E. coli* RcGTA g14 overexpression strains were run on an SDS-PAGE zymogram and each produced distinct zones of clearing in the incorporated SB1003 peptidoglycan (Fig. 6C), consistent with the idea that g14 is likely to be involved in enzymatically breaching the cell wall during infection. It is still unclear exactly how g14 may carry out this role; it would be expected that such a protein would form part of the RcGTA tail structure but proteomic analysis of purified RcGTA particles did not detect any peptides matching p14 [53]. The most probable explanations for this are that the p14 protein was either present but not detected, or that p14 is secreted from cells without association with RcGTA particles. Addition of concentrated, purified p14 protein directly to cells did not result in lysis or any obvious abnormalities (data not shown), and so the exact function of p14 is unclear.

In conclusion, RcGTA production in wild type cultures is a result of a substantial increase in expression of the RcGTA gene cluster in a small but distinct subset of cells, and this effect is magnified in overproducer cultures. This subset of cells may arise from a stochastic process, as described for the *E. coli lac* operon [54], or from a high frequency genetic change as in phase variation [55]. The mechanism for RcGTA release is likely to be via a lytic burst, because accumulation of extracellular RcGTA particles was concomitant with the appearance of intracellular components in the cell-free culture supernatant. In addition, a candidate gene annotated as a cell wall protease is not involved in lytic release of RcGTA but does play a role in invasion during infection of a target cell, perhaps by host recognition or through enzymatic digestion of the cell wall.
